# Surgical Cases Treated at Buffalo General Hospital.—Service of Dr. C. C. F. Gay

**Published:** 1881-10

**Authors:** C. C. Fredericks

**Affiliations:** House Surgeon


### Clinical Report of Surgical Cases treated at Buffalo General Hospital.—Service of Dr. C. C. F. Gay.

Reported by C. C. Fredericks, M. D., House Surgeon.

### COMPOUND FRACTURE OF SKULL—TREPHINING.

John Rick entered the Hospital March 21, 1881, aged 18; single; German; laborer. Two hours previous to entrance the patient, while attempting to jump aboard a train in motion, fell; the step of a car striking him on the back of the head. Patient complained of no pain, and showed no symptoms of concussion or compression. A slight scalp wound existed midway between the occipital protuberance and the mastoid process on the right side, and there was evident depression of the outer fragment of the occipital bone, the fracture extending downward toward the base of the skull.

Although there were no bad symptoms, Dr. Gay thought it advisable to elevate the depressed bone for fear of bad consequences in after-life. The fracture was found to be angular in form with one-eighth inch depression. A small button of bone was removed from the edge of the solid bone by the trephine, and the depressed fragment elevated. A spiculum of the inner table, half inch long, was found driven into the dura mater, and a small clot outside of the membrane at this point. Free drainage allowed. No brain symptoms supervened and April 25th patient was discharged well, the wound closed. Up to date

(June 1st) patient has been well and has returned to work. Pulse before and after the operation, 80; temperature, normal. Before operation patient protruded the tongue in a sluggish way. Pupils dilated. Result showed wisdom of not waiting for symptoms to develop.

### COMPOUND FRACTURE OF SKULL—OPERATION FOR TREPHINING.

Charles Scully, aged 23; single; born in Ireland; sailor. Entered the Hospital May 29, 1881. Two hours previous to entrance he had fallen seventeen feet into a dry dock, fracturing the radius of the right fore-arm and also producing a fracture of the frontal bone in the left supra-orbital region, with depression. Small scalp wound. No symptoms of concussion or compression appeared. June 3d, Dr. Gay cut down upon it and found the bone fractured in two directions, namely, one fracture in a vertical and the other a horizontal direction. The principal line of fracture being downward toward the eye. The trephine was used, a button of bone removed, and the slightly depressed bone was elevated, finding a small clot upon the membranes. Closed the wound partly, allowing free drainage; keeping the wound open by a tube; discharged well July 19. No bad symptoms followed the operation. The day before the operation the pulse was 60 per minute; left pupil fully dilated; the right pupil slightly dilated. The following table gives the record of pulse and temperature on the day of and subsequent to the operation:

| | Pulse. | Temperature. |
|---|---|---|
| June 3 | 56 | 100¾ |
| " 4 | 68 | 99¼ |
| " 5 | 62 | 99¾ |
| " 6 | 74 | 101½ |
| " 7 | 72 | 99¼ |

### FRACTURE OF THE LUMBAR VERTEBRÆ—DEATH—AUTOPSY.

George Henshaw, aged 40; married; born in England; laborer. Entered the Hospital March 23d, 1881. Three hours previous he was thrown backward by a falling partition against the edge of the wall of a fire pit, striking the wall across his lumbar region. On entrance only a slight displacement, a prominence of the third lumbar spinous process, was noticed. Patient complained wholly of pain in his back; had no power over his legs and only a little sensation; being able to tell when pricked or touched only. Patient was placed upon his back and extension applied, 25 lbs. to each leg, making counter-extension by raising the foot of the bed. Patient had from the first no control of bladder or bowels, requiring catheterization three times daily and warm salt water injections into the bladder. Cystitis soon developed, with not much pain, but ammoniacal urine and great quantities of mucous. A large slough found at the point of injury over sacrum and lower vertebræ, to which applied poultices. At the end of a week he was hung up and a plaster-jacket applied, which gave him a great support and consequent relief from pain, but, of course, no restoration of the power of sensation or motion. Patient gradually failed, and died April 19, 5.40 A. M.

AUTOPSY ON SAME DAY.—Found the spinous process and articular processes of the third lumbar vertebra broken, with some displacement of the body forward, the inter-vertebral cartilage being torn between it and the second lumbar vertebræ. The cord disorganized at the point of fracture and softened above and below.

SYPHILITIC PARALYSIS—DEATH—AUTOPSY.

Myra Bell, entered the Hospital April 12, 1881; aged 20; single; American. Three weeks before entrance patient had an attack of partial paralysis, but under iodide of pot. gr. xx *ter die* she got better and was up. Two days before entrance she was again seized with a more severe paralysis of the right side, unable to speak or swallow without great difficulty, and insensible to pain. Patient's friend gave her a syphilitic history, she having had a venereal sore three years, before followed by cutaneous eruption, sore throat, periosteal pain, etc.

Ordered by Dr. Gay, iodide of pot. gr. xxx, three times daily; protoiodide of mercury gr. ½ every six hours, all the milk and beef-tea she could take. April 15, unable to swallow at all, hence, ordered enemata every four hours of egg-nog, beef-extract and iodide pot. gr. xxx. Patient sank gradually and died April 18, 4.30 A. M. At no time had the patient a temperature above normal. Autopsy same day, 4.30 P. M. Heart, lungs, liver, kidneys, etc., all normal. Brain and membranes congested, with a deposit of lymph beneath the pia mater over the whole superior curved surface of the hemispheres, also a large clot of blood on the left side into the substance and between the convolutions of the greater part of the posterior lobe of the cerebrum. No effusion into the ventricles.

